# Research on 18th-Century Building Structures in Terms of Static Scheme Changes

**DOI:** 10.3390/ma16247689

**Published:** 2023-12-18

**Authors:** Monika Mackiewicz, Janusz Ryszard Krentowski, Kamil Zimiński, Aldona Skotnicka-Siepsiak

**Affiliations:** 1 Faculty of Civil Engineering and Environmental Sciences, Bialystok University of Technology, Wiejska 45E, 15-351 Bialystok, Poland; janusz@delta-av.com.pl (J.R.K.); kamilziminski@op.pl (K.Z.); 2 Faculty of Geoengineering, University of Warmia and Mazury in Olsztyn, Heweliusza 4, 10-724 Olsztyn, Poland; aldona.skotnicka-siepsiak@uwm.edu.pl

**Keywords:** historic buildings, structural element damage, technical condition assessment, changes in the static scheme

## Abstract

The evaluation of the technical condition of historic buildings that have operated for several hundred years is a complicated issue. Even buildings that are in very poor condition must be checked and assessed in terms of their further repair, strengthening, or compliance with conditions that allow the facility to be safely operated. Most 18th-century buildings have not survived to this day retaining their original arrangements and structural elements. Renovations and repair work in the past were often carried out using materials of uncertain quality, with repair work of different qualities and without detailed analysis or methodology, based only on the experience of the former builders. In historic structures, the character of the work of individual structural elements has often changed due to significant material degradation, the poor quality of repair work, or the loss of adequate support. When load transfers change, internal forces are redistributed, and, as a result, the static scheme changes. This article presents an overview of identified defects affecting the change in static schemes in historical building structures built in the 18th century, using the example of a historic building with a large number of aforementioned defects. The process of assessing the technical condition of the facility is presented, in which non-destructive testing (NDT) methods were used. Detailed computational analyses were carried out for the wooden roof truss structure, which had partially lost its support.

## 1. Introduction

The assessment of the condition of historic buildings must be carried out according to a specific schedule and in accordance with relevant regulations [1]. The choice of repair method and type of structure strengthening [2,3] may apply both to historic buildings [4,5,6]—which can be several hundred years old—and to buildings erected relatively recently but damaged as a result of the progressive degradation of structural elements, exceptional loads caused by fire or war, errors in construction work, or low quality of building materials [7,8,9]. Historic buildings are a source of knowledge about the building technology of former times and, therefore, as symbols of cultural heritage, must be legally protected. Therefore, it is necessary to monitor and check their technical condition and to protect and appropriately strengthen them [10]. The poor conditions of historic buildings or the high costs of their revitalization do not qualify them for decommissioning and possible demolition. Due to their cultural heritage, they must be carefully assessed in order to be approved for safe operation.

Traditional methods for identifying defects and assessing their impact on the character of structural work may turn out to be unreliable when the condition of facilities that have operated for several hundred years is checked [11,12]. Conducting destructive tests in historic buildings requires obtaining appropriate permissions so that the number of destructive tests is limited to the necessary minimum. However, NDT methods remain, requiring people with an appropriate level of experience to carry out this type of testing. NDT using modern devices allows for a much easier diagnosis. For example, georadar, sonic and radar tests [13], and ground-penetrating radar (GPR) [14] enable the detection of internal defects in structural elements. Terrestrial laser scanning (TLS) is also a useful tool for architectural investigations of the external and internal structures of buildings [15,16]. TLS enables not only the accurate reflection of architectural details of the façades of historic buildings [17] but also, using reflection intensity, allows for the analysis and determination of the material from which a given structural element is made [18].

Examples of testing procedures using NDT or semi-destructive techniques (SDTs) are popular in the literature [19,20,21], especially due to increasingly widespread access to devices enabling this type of analysis [22,23]. Numerous described analyses and examples of specific studies [24,25] also confirm the validity of using these methods in historic buildings. NDT used in the assessment of historic buildings concerns both masonry structures and wooden structural elements [24,26,27,28].

In addition to the research and assessment of the actual condition of historic buildings, issues relating to the impact of harmful external factors on the properties of materials from which the structure was made have also been analyzed [29,30]. A useful tool for assessing the technical condition of historic buildings is historic building information modeling (HBIM) [31,32]. Multidisciplinary methodologies are used to implement building information modeling (BIM) for the analysis of historic buildings [3,5]. Taking into account a BIM flowchart supports the process of assessing the condition of the structure, as confirmed in [33,34]. Aspects taking into account sustainable development are also important during the process of condition assessment and renovation [35].

All the above-mentioned methods and tools used in the analysis of technical structural conditions enable the undertaking of a rational decision regarding potential repairs and strengthening [2,3]. Attention and care to the preservation of the original architecture, historical form, and detail requires a comprehensive approach in this scope [36]. The essence is the awareness that any interference in a material structure is intended to extend the time of safe operation of the building structure. This is why structural testing after the strengthening and repair stage plays such an important role [37]. When designing new structures and assessing the condition of structures that have been in use for several decades, it is important to meet technical regulations and the required level of safety [38,39,40,41]. In historic structures, even after strengthening, it is often not certain that safety reserves are at the required level. Therefore, more useful tools are being developed to support the process of solving problems relating to design, planning, management, erection, and operation processes. New solutions and ideas that solve technical and technological problems, which may appear in every single stage of projects, must be substantiated. The methodology of TRIZ (theory of inventive problem solving) may be a useful tool in this aspect [42]. This also applies to replacing existing materials while taking into account an effective eco-design strategy [43].

This paper presents an important problem, namely, changes in the load transfer and the redistribution of internal forces in 18th-century building structures. Examples of defects, damage, and human interference observed in historic buildings, affecting changes in the static schemes of structural elements, are described. A significant number of analyzed defects were observed in a historic pavilion building of a historic palace complex located in the northeastern part of Poland. Using this building structure as an example, not only were the existing defects described but numerical analyses concerning the impact of defects on static scheme changes of wooden roof truss were also carried out.

One of the problems discussed in this paper is the analysis of the wooden roof truss structure. The analysis and assessment of historic wooden structures were described in the literature regarding similar issues. Examples that describe the methodology for assessing the condition of historic wooden structures are [44,45], where in both cases, NDT methods were used. Correlations in the NDT tests, visual grading, and destructive tests were undertaken in [46]. In the work [47], the relationship between the results obtained from NDT and destructive tests was described by emphasizing how important it is to identify wood species. In this paper, the use of NDT methods to determine the material parameters of wooden elements with the highest possible accuracy is described. Moreover, the analysis using numerical calculations is presented. This article describes an attempt to determine the factors influencing the changes in the static scheme of the structures. This is what distinguishes analyses presented in this paper from previous contributions.

## 2. Defects Resulting in Static Scheme Changes in Structure

An important aspect of assessing a building’s condition is to identify defects and determine the causes of negative destruction processes. In addition to the natural aging processes of structures, phenomena that accelerate the degradation of the facility often occur. However, it should be remembered that the main reason for the deterioration of the technical condition of buildings is the lack of ongoing repairs and proper facility maintenance by the owners. Historic buildings are under official conservation protection, which effectively prevents this. There are a number of destructive methods that allow for the determination of current material parameters. In historic buildings, carrying out such tests requires special permits. Therefore, whenever possible, NDT methods are used. An additional issue that may arise during the research and analysis of a structure’s condition is the possibility of change in the static scheme. There can be several reasons for changes in static schemes. The most important are degradation of construction materials, excessive settlement of supports, excessive looseness in joints, and changes in the geometry of structural elements (change in dimensions, deflections due to changes in humidity, shrinkage, and rheological phenomena). Geometric changes in structural elements may occur in the elastic and inelastic range. Geometric changes in the inelastic range may be caused by rheological effects, brittle fracture or plasticization, biological and chemical corrosion, physical processes such as cyclic freezing and thawing, excessive moisture caused by leaky roofing or faulty ventilation [48,49], and washing out or dissolving small particles from the material structure. Presented below are examples of historic buildings from the 18th century in which, as a result of defects, changes in the static scheme of structural systems occurred.

### 2.1. Realization of Installation below the Foundation Level

An investigation of the destruction state was carried out on a historic three-story building dating from the second half of the 18th century, which was constructed as a traditional brick wall structure. In the building, there was no basement, and the foundation walls were made partly of cobblestone and full ceramic brick with lime mortar.

Installing pipes, for example, during the modernization of the sewerage network, close to buildings and below the level of foundations is associated with high risk. Deep excavation below the foundation level, without appropriate protection, results in changes in the natural structure of the soil. It may cause uneven settlement of the foundation and, consequently, cracking of load-bearing walls. In extreme cases, the installation pipe leaks, and the flowing water or sewage washes out small particles of soil under the foundations. This also leads to uncontrolled settlement in the foundations and cracking of the walls.

**Figure 1:**
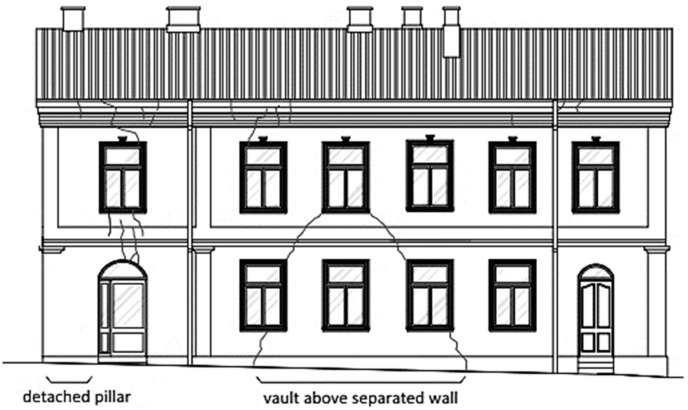
Separation of the wall fragments, due to scratches and cracks, into smaller parts that work independently.

In the building presented in Figure 1, the width of the existing cracks significantly increased and new cracks appeared just after the completion of earthworks related to the modernization of the installation connection to the building. An inventory of the wall cracks is presented in Figure 1. As a result of the cracks, the method of load transfer in the external longitudinal wall was changed. The wall was divided into smaller fragments, which began to function as separate independent elements, similar to pillars, arches, and vaults.

### 2.2. Eccentrically Constructed Walls and Collapsed Vaults

**Figure 2:**
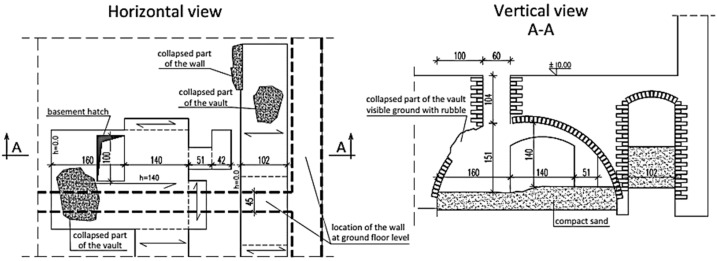
The structural arrangement of a basement vault from the turn of the 18th and 19th centuries.

Defects that cause changes in the static schemes were observed in a two-story building in which the basement walls were built at the turn of the 18th and 19th centuries. The above-ground walls were constructed in the second half of the 19th century, preserving the basement of the building that previously existed in this place. The arrangement of the basement walls does not correspond to the arrangement of the ground floor walls. The internal longitudinal load-bearing wall on the ground floor does not coincide with the basement wall and is based on the brick vault not on the wall located in the basement, as shown in Figure 2. The offset of the walls is approximately 90 cm.

The basement is partially filled with soil and rubble. In the place where the locally collapsed vault is located, part of the load-bearing wall on the ground floor has no support. Therefore, a natural secondary vault was created in the wall over the damaged vault. The load transferring was changed, and the forces were transmitted through wall fragments with appropriate support on the brick vault.

### 2.3. Destructive Impact of Rainwater

Leaking gutters and downpipes cause improper drainage of water from the roof, which results in dampness and subsequent degradation of the walls. In the long term, it may cause serious damage to the walls and foundations. Moisture in the walls is particularly dangerous in temperate climates in the winter when the temperature outside is around zero. Water freezes up and thaws out repeatedly in the wall cracks and pores of ceramic elements.

**Figure 3:**
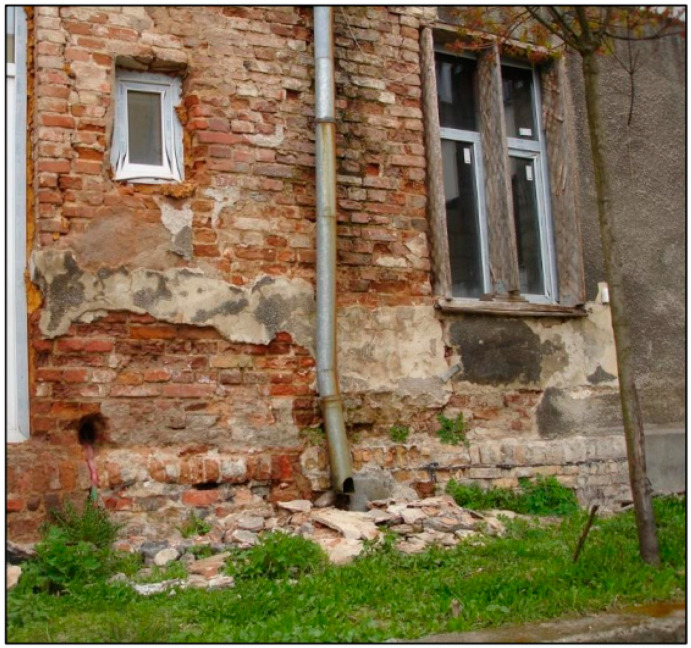
Damage to a plaster and brick wall due to leaking drainpipes.

The result is visible in the form of plaster separations and the defects propagations, Figure 3. Bricks and mortar are damaged. The thickness and the load-bearing capacity of the wall is reduced. Vertical forces from higher floors are displaced in relation to the axis of the foundation walls, which additionally influences their uneven settlement.

Draining rainwater into the ground close to the foundations, especially shallow foundations in non-cohesive soil, leads to the washing out of small particles from the soil framework. As a result, the soil structure is weakened, and the foundations settle. This is a common cause of cracks and scratches on the walls.

### 2.4. Consequences of Wall and Ceiling Reconstruction

In a three-story residential building from the end of the 18th century, numerous reconstructions were carried out during the building’s operation. The originally homogeneous brick walls were transformed into separate parts with different technical conditions and strengths. Moreover, in the same building, during the reconstruction, wooden ceilings were demolished and replaced with reinforced concrete ceilings on steel beams. Ceiling slabs only provide lateral support for walls if they have adequate stiffness. The demolition of ceilings on several levels at the same time, leaving only wooden ceiling beams without sheathing, led to a change in the stiffness of the lateral support of the walls. When the sheathing was dismantled, the ceilings lost their stiffness and stopped providing continuous support for the walls. As a consequence, until new ceilings were constructed, there was a change in the method of lateral support and buckling length of the walls.

### 2.5. Construction of a Ceiling with Low-Stiffness Beams

**Figure 4:**
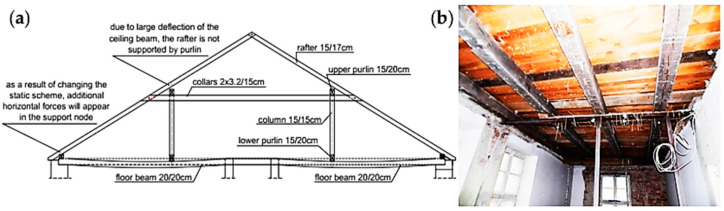
Deformation of a ceiling with wooden beams: (**a**) roof truss structure supported on the deformed ceiling and (**b**) view of the ceiling temporarily supported in order to limit the deflection.

An interesting case is a single-story production building from the end of the 18th century, which is covered with a gable roof supported with longitudinal walls and a wooden beam ceiling. During modernization work in the second half of the 20th century, thermal insulation was placed on the ceiling and a 7 cm thick floor of cement screed was made on it. The additional load on the ceiling increased the deflection in the ceiling beams, as shown in Figure 4a. Therefore, the ceiling structure required temporary support, as shown in Figure 4b. During repair work in 2022, all floor layers were removed from the ceiling. It did not completely eliminate the deflection in the ceiling beams. The beams remained permanently deflected due to rheological effects.

The construction of ceilings using beams with insufficient stiffness leads to excessive deflections. The problem starts when other structural elements, e.g., a roof truss, rest on excessively deflected ceiling beams. The excessive deflection in the beams changes the support and distribution of internal forces in the structure based on such a ceiling. This leads to changes in the static scheme.

## 3. Analysis of the Destructive Stage of a Pavilion Building

**Figure 5:**
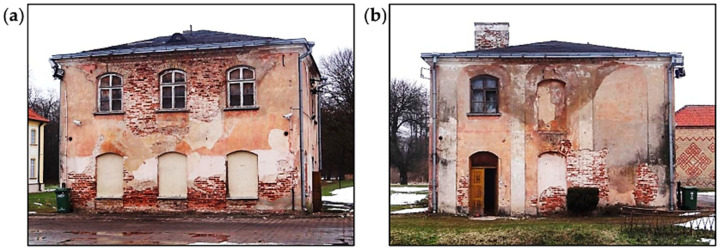
Pavilion building: (**a**) front elevation and (**b**) side elevation.

The accumulation of defects and damages, which contribute to static scheme changes, can be presented in the example of an 18th-century pavilion building that is a part of the historic palace complex, as shown in Figure 5. During the assessment of the facility condition, the effects of significant degradation of individual structural elements were observed. The degradation of roof truss elements and the loss of proper support contributed to a change in the character of the work of the roof truss structure. In the analyzed case, as a result of the tests performed, the degree of collapse risk of the roof structure was assessed, which was particularly important for the planned renovation work.

This section describes a number of defects found in the structural elements of the pavilion building. The description of defects is preceded by a presentation of the history and the structure of the building.

### 3.1. History of the Pavilion Building

The foundations of the pavilion building were laid in 1753. The construction and finishing work took several years. After 1840, the pavilion was intended to serve as a residence for factory officials. In 1915, during World War I, the palace buildings were partially blown up, and only the one-story walls of the pavilion building. remained In 1937, the palace buildings were rebuilt and used as a hospital. During World War II, the hospital was closed down, and the Soviet army was quartered in palace complex buildings. After 1941, a camp for several thousand prisoners of war was organized. After the end of the war, the hospital was reactivated, and the pavilion building was used for the needs of the hospital farm until 2003. There were offices on the ground floor, and the first floor was residential. Currently, the pavilion building is not in use. During the last 20 years, the condition of structural elements has been progressively degrading. The current arrangement of the main structural elements is described in the next section.

### 3.2. Building Structure

The pavilion building was constructed as a two-story building, without a basement, with an attic and covered with a hipped roof made as a wooden structure with traditional technology, as shown in Figure 5. The walls were made of solid ceramic bricks. The ceiling above the ground floor was made of reinforced concrete slabs, and the ceiling above the first floor was made entirely of wood with wooden beams. The roof truss was made of wood in a rafter–purlin structure. The ceilings and roof truss were built in 1937.

The foundation of the pavilion building was made of cobblestone with lime mortar. The lintels were made of arched brick. Some of the lintels, built during the reconstruction in 1937, were made of steel beams. The building was rebuilt several times. The effects of numerous brickworks and changes in the location of existing window and door openings were identified on the external walls.

### 3.3. Building Defects

**Figure 6:**
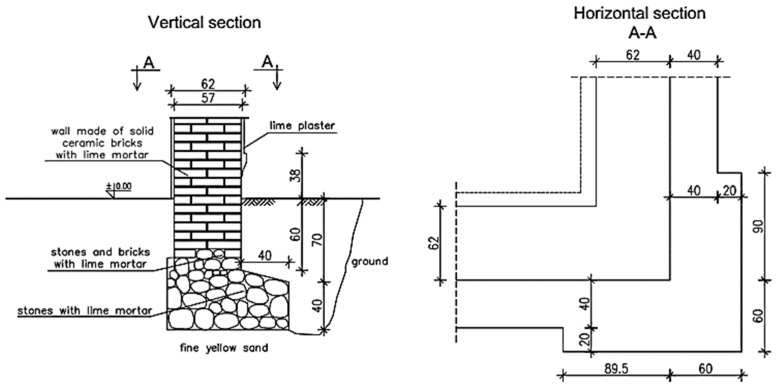
Vertical and horizontal sections of the foundation extension in the building corner.

The process of building condition assessment began with the foundations, which were made of cobblestone arranged in a masonry bond manner. Based on the excavations, it was found that the foundation stone wall has a compact structure. There was no mortar in the joints on the upper surface of the foundations, but lime mortar in a satisfactory condition was found deeper in the joints. The foundations were wider than the foundation walls by approximately 40 cm, and the building corners by approximately 60 cm, as shown in Figure 6.

The foundation walls had the same width as the above-ground walls. Their bottom was approximately 60 cm below ground level. The upper part of the foundation walls was made of ceramic bricks with lime mortar, and the lower one was made of cobblestone and solid ceramic bricks with lime mortar. The mortar on the wall surface was degraded, and its strength was close to 0 MPa. Small scratches, which were caused by uneven settlement in the building, were found on the foundation walls just above ground level.

**Figure 7:**
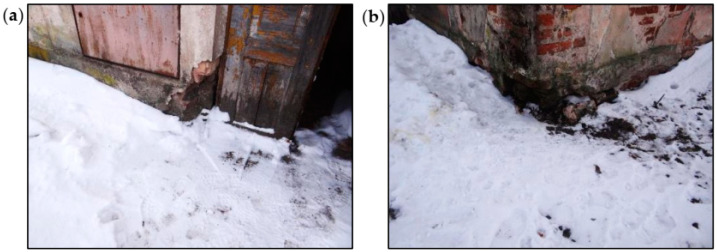
View of defects in external brick walls: (**a**) near a door opening and (**b**) in the corner of the building.

Defects in the walls aboveground were concentrated in the corners of the building, as shown in Figure 7. The bricks and lime mortar in the surface layers were significantly degraded in many places and had a strength close to 0 MPa. In the deeper parts of the wall, the mortar had a color typical of lime mortar, and its strength was estimated at approximately 0.2 MPa. The strength of bricks determined using destructive tests in the laboratory met the standard criteria.

**Figure 8:**
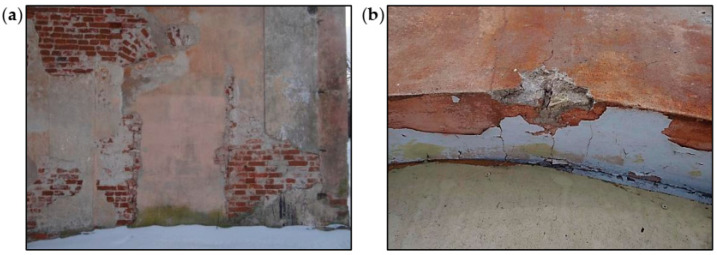
Damage to external walls: (**a**) plaster losses and bricked-up door opening and (**b**) crack in the arch above the window.

**Figure 9:**
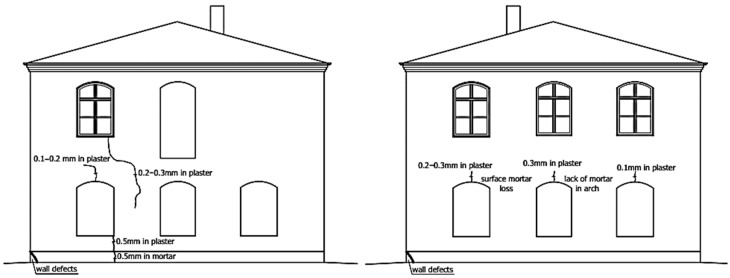
Inventory of scratches on two external walls.

The significant degradations of the brick walls and plaster losses visible on the surface of the building’s façade were observed. Above the windows in the arched lintels, damage was indicated in the form of scratches in the plaster and the loss of mortar, as shown in Figure 8 and Figure 9. The causes of damage to the brick arches above the windows and doors were uneven settlement of the foundations, deformations of the walls, dampness of the walls, and freezing and thawing of water inside the wall structure.

Large areas of the walls throughout the building were damp. In selected places, wall humidity measurements were performed using the electro-resistance method. The greatest dampness occurred at the ground level and just above it.

Water is a significant destructive factor in the case of brick walls. Capillary properties of the brick cause water to come up from the ground to the height of several meters of the wall, simultaneously transporting water-soluble salts. Salt crystallization in the surface layers of the bricks causes the mechanical destruction of clay material structure, resulting in crumbling and brick fragments falling off the walls. Salts, due to the hydrolysis process, change the reaction of water to slightly acidic or alkaline. This results in the destruction of mortar and the dissolution of clay material in bricks.

In the pavilion building, the effects of capillary phenomena were visible on the walls, especially on the lower parts. Low temperatures and water freezing led to mechanical corrosion of the walls. Locally, the corrosion reached a depth of several centimeters inside the wall.

In the walls with lower dampness, low temperatures also cause systematic separation of plaster coatings after subsequent winter periods. After the wall is exposed (the plaster is chipped off or it falls off on its own), the effects of brick corrosion are visible in the form of brick fragments that flake, crumble, and falling off.

There were also visible effects of the corrosion caused by rainwater, flowing down from the top of the walls. The capillary properties of bricks and mortars cause rainwater to be excessively absorbed in the places where the walls are damaged. In such places, due to the difficult evaporation of water, significant damage occurs when water freezes in the winter.

Another factor causing corrosion is the active growth of microorganisms in walls with increased humidity. As a result of algae growth, substrate degradation occurs due to the secretion of organic acids. Acidic products of algae metabolism change the chemical composition of the water in wall capillaries, which additionally increases corrosion.

Based on the analysis of external walls, the following defects and findings were noted: significant surface damage to the walls, local defects just above ground level, local scratches, relatively low compressive strength of the bricks (class 5), and relatively low compressive strength of the lime mortar (approximately 0.2 MPa). However, no significant vertical or horizontal deformations of the external walls were detected. There were also no cracks propagating through the entire width of the wall. Due to the above-mentioned factors, it should be stated that the technical condition of the external walls was sufficient.

**Figure 10:**
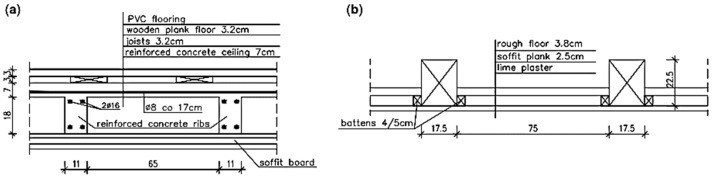
Ceiling sections: (**a**) concrete ceiling above the ground floor and (**b**) wooden ceiling above the first floor.

**Figure 11:**
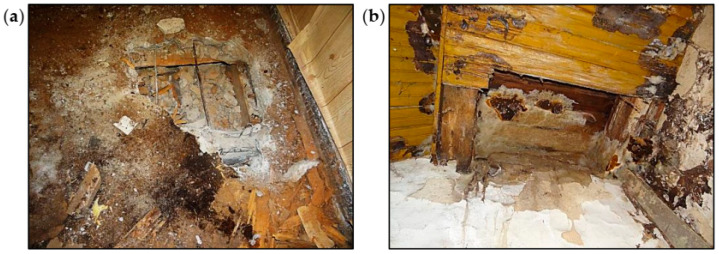
Ceiling opencasts: (**a**) from the top of the reinforcement concrete ceiling and (**b**) from the bottom of the wooden ceiling.

The ceiling above the ground floor was made of reinforced concrete slabs and ribs, as shown in Figure 10a. The ceiling was covered from below with a suspended ceiling made of cement-lime plaster on steel mesh. Between the ceiling and the ceiling ribs, there was a soffit made of boards. Two opencasts of the ceiling were made: from the bottom and the top (Figure 11a). Based on the opencasts, it was found that the ceiling concrete slab was made with a thickness of 7 cm, and the main reinforcement of the slab consisted of smooth bars with a diameter of 8 cm every 17 cm. There was no distribution reinforcement in the slab on a section of 40 cm. The ribs were 11 cm × 25 cm (height measured including the ceiling slab). In the ribs, no reinforcement was found in the upper zone, but just under the reinforced concrete slab, there were two bars with a diameter of 16 cm. In the ribs, no stirrups were found on a section of 30 cm.

Sclerometric tests showed that the ceiling was made of C8/10 class concrete. Due to the lack of stirrups in the ribs in the support zone, it should be considered that the shear load capacity of the reinforced concrete ribs was insufficient. Due to the lack of stirrups or their too large spacing in the ribs, the lack of distribution reinforcement in the ceiling slab, and the low-quality concrete, it should be considered that the condition of the reinforced concrete ceiling was poor.

**Figure 12:**
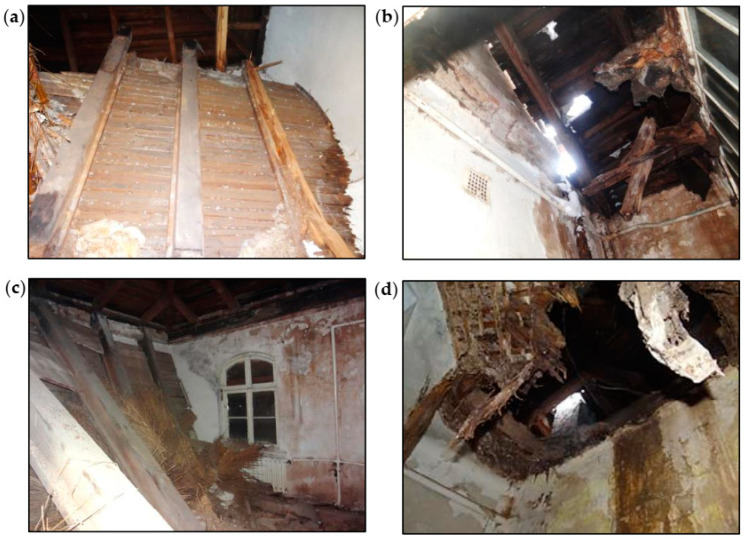
View of the collapsed wooden ceiling: (**a**,**b**) above the staircase and (**c**,**d**) above a room on the first floor.

The ceiling above the first floor was made of wooden beams, as shown in Figure 10b. Leaks in the roof covering caused dampness of the ceiling and contributed to the development of biological corrosion. The ceiling above the staircase and the room on the southeast side completely collapsed, as shown in Figure 12. The remaining parts of the ceiling were very damp, as shown in Figure 11b. There were plaster defects and cavities in the sections close to the collapsed parts of the ceiling. The ceiling beams were significantly damaged as a result of biological corrosion. The remaining parts of the ceiling were in danger of collapse. The overall condition of the wooden ceiling was poor.

Leaks in the roof covering caused dampness in the wooden elements of the roof truss, and the biological corrosion developed. Wooden elements close to the chimney eaves and a leaky roof covering were significantly damaged. Some of the rafters were damaged in the places where they rested on the roofing wall plates. The collapse of the wooden ceiling above the first floor caused the pillar walls of the roof truss to be supported only on the brick walls and fragments of the remaining ceiling. The pillar walls above the staircase and the room on the southeast side had no support.

## 4. Tests of Materials and Measurements Carried out during the Condition Assessment of the Pavilion Building

### 4.1. Destructive Testing of Bricks

**Figure 13:**
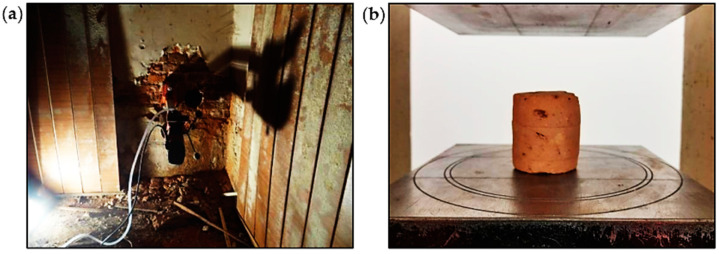
Destructive testing: (**a**) cutting out brick samples from the wall and (**b**) the strength test of a brick.

The quality of bricks in the walls was assessed based on laboratory tests of compressive strength. Drilled holes (Figure 13a) were made in the walls to collect samples for laboratory tests. During drilling, the mortar was washed out, which indicated that its compressive strength was equal to 0.1–0.2 MPa according to [50]. Strength tests of bricks (Figure 13b) were performed in accordance with applicable standards [51]. For three samples that were taken for testing, a total of 10 tests were performed. The average compressive strength of the individual brick samples was equal to 6.6 MPa, 7.9 MPa, and 4.7 MPa, as presented in Table 1. Based on the obtained strengths, the tested bricks should be classified as class 5, which is the lowest class of bricks. The strength of the lime mortar in the walls should be estimated at 0.2 MPa.

According to the findings obtained from destructive tests and taking into consideration the visual evaluation of the defects, the technical condition of the walls was classified as sufficient.

### 4.2. Wood Testing Using a Woodtester

During the condition assessment of the wooden roof truss, NDT methods were used such as measuring the wood density using a Woodtester and measuring the wood humidity using a moisture meter. The most important reasons for the poor condition of the roof truss were leaks in the roof covering, provisional repairs of defects, lack of proper flashing of the chimneys and gutters, lack of periodic inspections, and supplementary impregnation of the wooden truss structure with fungicides and insecticides. Humidity tests showed that the moisture content of the wooden elements of the roof truss was 30–50%.

The condition of the roof truss structure was thoroughly assessed visually. Progress of the biological corrosion and damage to the elements were very significant.

**Figure 14:**
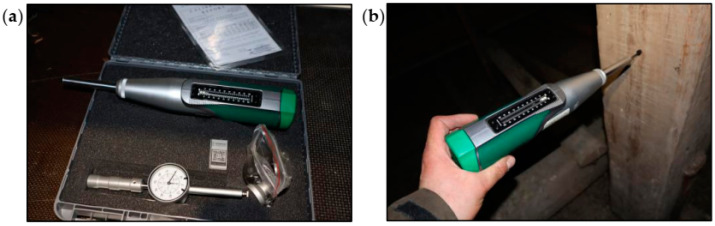
Woodtester: (**a**) view of the device and accessories and (**b**) during the test.

Despite the difficult access to the roof truss elements, due to the ceiling collapse, partial tests were performed using a Woodtester, as shown in Figure 14. Based on the depth of needle penetration into the wood, it was possible to estimate the density of the wood and then the average modulus of elasticity. In the case of the analyzed roof truss, the degradation of the cross-sections of the elements was significant. Based on the Woodtester tests, the average modulus of elasticity of the wood was estimated and applied in numerical calculations.

### 4.3. Terrestrial Laser Scanning of the Pavilion Building’s External Walls

Another NDT method used during the assessment of the pavilion building condition was TLS, which allows for performing comprehensive measurements of a building’s geometry. As a result, a point cloud is obtained, where each point has four coordinates assigned to it. Three of them, X, Y, and Z, are shown in the local coordinate system of the scanner and can be transposed to any geodetic system. The fourth coordinate describes the reflection intensity of the laser beam from the measured element. Scanning devices can collect data from a maximum distance of 300 m, with a horizontal field of view up to 360 degrees and a vertical plane up to 270 degrees. Measurements are performed with an accuracy of up to 1 mm.

TLS technology allows for performing analyses in the field of inventory of the actual shape and current condition of building structures as well as measurements of displacements and deformations in structural elements and buildings. A laser scanner is a useful tool for collecting geometrical data about the technical condition of building objects. Based on the measurements, it provides data on the shape and dimensions of the analyzed object. At the same time, defects in the form of deformations, cracks, or other surface damage are inventoried. The data obtained during the measurements are processed using computer programs, which allows for obtaining a virtual, spatial copy of a measured building [15,16,17,18]. Additionally, thanks to digital photos taken during the measurement, a three-dimensional model can be viewed in natural colors.

The 3D laser scanning and specialized computer programs allow for more complete, more accurate, and more legible documentation of the building. The obtained information is very detailed, so the scope of its application is wide. For this reason, scanners are increasingly replacing other measuring tools.

**Figure 15:**
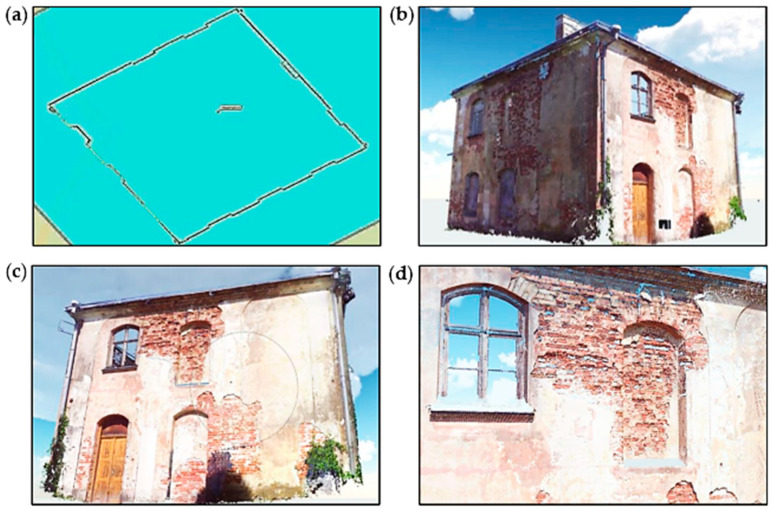
Images obtained based on the point cloud: (**a**) horizontal projection with building dimensions; (**b**,**c**) a view of the external walls and (**d**) a view of defects in the brick wall.

In the case of the pavilion building, laser scanning was performed. External walls were scanned, and the digital point cloud was obtained. Views of the pavilion building after the appropriate processing and development of the point cloud are shown in Figure 15. Thanks to precise measurements, the actual geometry of the pavilion building was available, as shown in Figure 15a. Significant advantages include the possibility to observe in detail the defects in external brick walls and also to determine the depth of these defects, as shown in Figure 15d.

## 5. Numerical Analysis of the Wooden Roof Structure of the Pavilion Building

**Figure 16:**
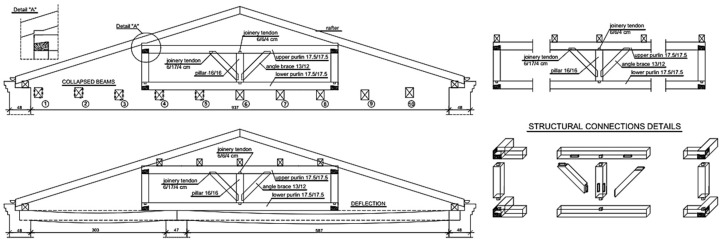
View of the roof truss structure supported by a partially destroyed ceiling.

The intensive degradation process of the wooden roof structure began with damage to the roof covering. It directly caused considerable dampness of the upper parts of the external walls, the roof truss, and the wooden ceiling above the first floor on which the roof truss was supported. The wooden roof truss and ceiling were attacked by fungi and were seriously damaged. The rapid progress of biological corrosion weakened and damaged the support zones of the ceiling beams. This resulted in the collapse of the ceiling parts (beams 1–5) supporting the roof truss, as shown in Figure 16. The roof truss structure partially lost its support. Despite serious damage, the roof truss did not collapse and remained in a state of unstable balance.

A spatial computational model of the roof truss structure was prepared in order to precisely assess the degree of effort of structural elements. Based on the Woodtester results, taking into account the actual material properties and rheology of the wooden elements, the average final modulus of wood elasticity was estimated as E_mean,fin_ = 3667 MPa [41]. The flexibility of the roofing wall plate support in the horizontal direction of 500 kN/m was assumed for all calculation variants. The loads were determined according to [38,39,40].

In the numerical analysis, computational models of the roof truss structure were made taking into account:

- The low value of the elastic modulus of the wood;
- The influence of humidity and rheology;
- The nature of the load and duration of the load (DOL) effect;
- The flexibility of supports in the horizontal direction (support on roofing wall plates);
- The degradation of some structural elements;
- The lack of supports due to the collapse of the wooden ceiling;
- The impact of deformation in the main structural elements on the secondary elements based on them;
- The character of the joints’ work (some joints can only transfer compressive forces);
- The discontinuities (looseness) in the joints.

**Figure 17:**
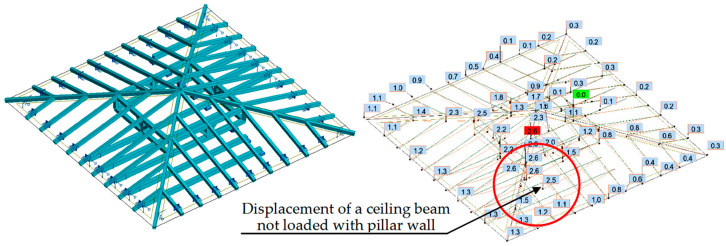
Variant I—model and displacements due to permanent loads.

In the first case (variant I), the calculations showed that the ceiling beam not loaded with the pillar wall had a greater deflection value than the adjacent ceiling beam loaded with the pillar wall. The displacement diagram showed that the pillar wall was not supported on the ceiling beam, but the ceiling beam was suspended to the pillar wall. Observation of this phenomenon was possible for permanent loads acting on the structure, as shown in Figure 17. Since the loads transferred from the roof were relatively small, tensile forces occurred in the connection of the ceiling beam with the pillar wall, which was inconsistent with reality.

**Figure 18:**
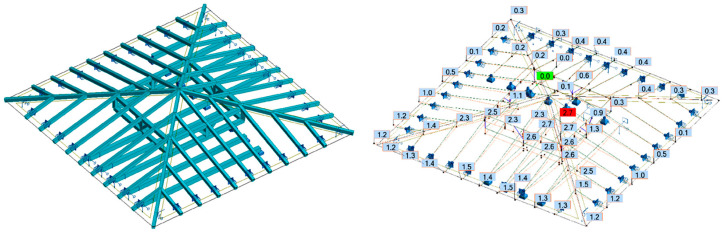
Variant II—model and displacements due to permanent loads; the pillars and swords can transfer only compressive forces.

In the second case (variant II), it was assumed that the pillars and swords could not transfer tensile forces due to the character of the work of the carpentry joints. For the same permanent loads, the deflection in the ceiling beam supporting the pillar wall was greater than the deflection in the adjacent ceiling beam not loaded with the pillar wall, as shown in Figure 18.

**Figure 19:**
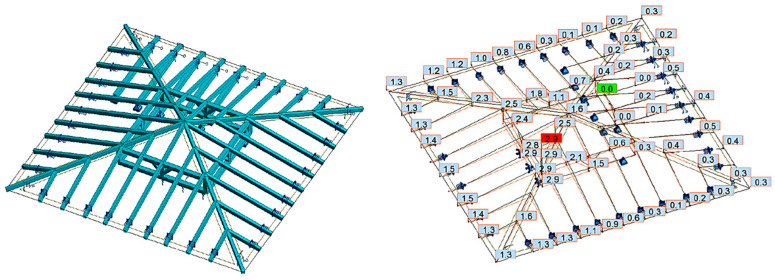
Variant III—model and displacements due to permanent loads; the pillars and swords can transfer only compressive forces; and flexible supports with the initial deflection of the ceiling beams.

In the third case (variant III), the same permanent loads and the same assumption that the pillars and swords can transfer only compressive forces were applied. The modification assumed that the supports of the roof truss in the form of the remaining ceiling beams were replaced with elastic supports with flexibility corresponding to the ceiling beams. Moreover, the displacements in the support points were assumed to be equal to 2.5 cm to correspond to the deflection in the ceiling beam under the ceiling’s own weight, as shown in Figure 19.

**Figure 20:**
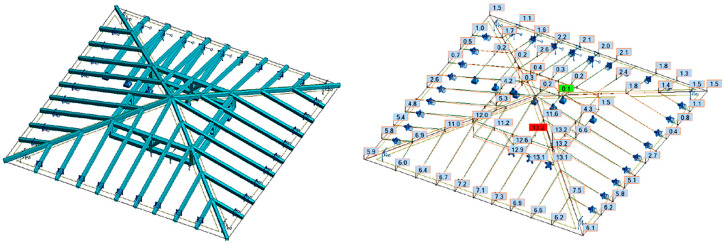
Variant IV—model and displacements due to permanent loads and snow, pillars, and swords can transfer only compressive forces and flexible supports with the initial deflection of the ceiling beams.

In the fourth case (variant IV), calculations were made using the same assumptions as in variant III. In addition to the permanent loads, the loads from snow were also taken into account, as shown in Figure 20.

In the presented diagrams, different values for displacements in the pillar wall were obtained. It mainly depended on the method of support and the possibility of transferring tensile forces through the pillars and swords. Based on the analyses and calculations performed, it was confirmed that the spatial computational model of the roof truss is susceptible to many factors, which have a significant influence on the displacement results.

If the stiffness of the supports (in this case the ceiling beams) was low, the first calculations showed that the roof truss was not supported by the ceiling beams, but the ceiling beams were suspended to the roof truss, which is inconsistent with reality. It was necessary to analyze the character of the work of the carpentry joints. On this basis, an assumption was made that some elements of the roof truss were unable to transfer tensile forces. These were the elements that were connected with the other elements with carpentry joints. After taking this assumption into account in the calculations, the internal forces and displacements in the structure changed significantly.

## 6. Discussion

All defects described in this article have a negative impact on the technical condition of structural elements. The destructive impact of external factors can be divided into two groups:

- Group 1—destructive processes cause a gradual deterioration of the strength parameters of structural elements until their load-bearing capacity is reached.
- Group 2—destructive processes, at some stage, cause a change in the static scheme, as a result of load-bearing capacity loss of a single or several elements or as a result of excessive deformations in individual structural elements.

In the brick walls, making additional openings or filling existing openings with non-structural materials causes several fragments of walls may be not connected to each other and to work independently. As a result of uneven settlement of the foundations, cracks in the walls appear. Natural dilatations are created and individual wall fragments work independently of each other. Above the separated fragments, the wall can maintain its continuity, e.g., by creating a spontaneous secondary arch.

In the case of wooden structures with carpentry joints, the distribution of forces and stresses is more difficult to interpret. As a result of the redistribution of internal forces and change in the static scheme, the structure may collapse or maintain spatial stiffness and still continue the safe transfer of loads in the new static scheme.

During the condition assessment process of historic buildings, it is necessary to conduct a detailed analysis, taking into account as many of the previously mentioned factors as possible. The analysis of masonry structures should consider, among others, wall re-building, the variety of materials from which parts of the wall were made, ground conditions, etc. In the case of spatial roof truss structures, as a result of progressive degradation, damage propagation, and reduced stiffness of structural elements, a change in the static scheme may occur. As a result, tensile forces appear in elements that previously had compressive forces. If the carpentry joints are unable to transfer tensile forces, a secondary change in the static scheme occurs. For the computational calculations, it is necessary to assess whether the displacements in the structure are consistent with the actual situation. It should also be checked whether the obtained internal forces can actually occur. Due to the character of the work of the supports and joints, elements have a limited ability to transfer various types of forces. During the computational analysis, carpentry joints in traditional wooden structures should be carefully examined because, depending on their type, they can transfer only compressive forces and cannot transfer tensile forces.

Multithreaded analyses are always challenging, despite the considerable experience of those who perform them. One of the most important repercussions, resulting from the conducted research, is a statement that the identification of the appropriate static scheme is often a difficult and demanding task. Errors may appear in the form of oversimplifications of computational models and material parameters. Appropriate methodologies and tools should be used to support the assessment process, especially in order to notice changes in the static schemes of the structure. The main implication of this study is the confirmation of the validity of using NDT methods in the condition assessment process.

## 7. Conclusions

Research and analyses of the structures presented in this article lead to the conclusion that many processes and actions related to progressive wear, changes in use, repairs, and reconstructions lead to changes in static schemes. Some of them may have a local impact, but often, the changes affect the static work of almost the entire structure. Obvious situations are removing or changing the flexibility of the supports and changing the stiffness of structural members and connections.

The decrease in the strength properties of building materials is caused by:

- Corrosion and anthropogenic processes;
- The aging of materials;
- Changes in loads acting on the structure;
- Changes in environmental conditions (temperature, humidity).

These factors cause the loss of load-bearing capacity of individual structural elements. In extreme cases, it results in failure, or the structure remains stable, but redistribution of internal forces and changes in the static scheme occur.

Taking into consideration aspects such as the impact of rheological phenomena, the effects of DOL, humidity, temperature, the actual technical parameters of materials, including degradation, and the character of joints is important in the process of assessing the condition of degraded historic building structures. All defects in structures contribute to changes in the static schemes. Based on the research presented in this article and the results obtained, the possibility of changes in the static schemes should be considered. This aspect requires emphasis because it has not been strongly promoted in the cases of the condition assessment of structures, which were previously described in the literature. The main limitation is the lack of dedicated guidelines for this purpose. However, research and the application of different methods are a part of the expected future development.

## Figures and Tables

**Table 1:** Compressive strength of brick samples obtained from laboratory tests.

Sample Number	Core Diameter (mm)	Maximum Compressive Force (kN)	Strength (MPa)	Average Value of Compressive Strength (MPa)
1.1	54	9.9	4.3	6.6
1.2	54	18.9	8.2	
1.3	54	11.0	4.8	
1.4	54	20.6	9.0	
2.1	54	20.8	9.1	7.9
2.2	54	11.6	5.1	
2.3	54	21.6	9.4	
3.1	53	10.9	4.9	4.7
3.2	54	12.3	5.4	
3.3	53	8.4	3.8	

## Data Availability

The data presented in this study are available on request from the corresponding author.
